# Fractional Laser-assisted Hair Regrowth and Microneedling for the Treatment of Alopecia Areata: A Review

**DOI:** 10.7759/cureus.4943

**Published:** 2019-06-19

**Authors:** Robert J Dabek, Daniel S Roh, Derman Ozdemir, William G Austen Jr., Branko Bojovic

**Affiliations:** 1 Plastic Surgery, Massachusetts General Hospital and Harvard Medical School, Boston, USA; 2 Internal Medicine, Saba University School of Medicine, The Bottom, Caribbean Netherlands, NLD

**Keywords:** alopecia areata, fractional lasers, lasers, microneedling, hair loss, laser-assisted drug delivery

## Abstract

Alopecia areata (AA) affects approximately 2.1% of the population, with women being affected more often than men. Current therapies consisting of topical corticosteroids or intralesional injections are often the first choices for treatment, but are limited by unsatisfactory outcomes or risks to patients. Recently, fractional lasers and microneedling, with or without the addition of topical agents, have been examined as treatment options. A literature review was performed to evaluate the efficacy of fractional lasers in the treatment of AA. A total of six fractional lasers and two microneedling studies consisting of small prospective and retrospective studies, and case reports were reviewed. The number of trials and participants are limited, but evidence suggests that fractional lasers and microneedling may be effective therapeutic approaches when coupled with topical agents. Larger studies are required to better understand the effects of these treatment modalities for AA.

## Introduction and background

Alopecia areata (AA), an autoimmune condition, characterized by a chronic remitting-relapsing course of patchy hair loss, affects approximately 2.1% of the population [1]. Adults and children are affected, with women being at slightly higher risk [1]. It is estimated that approximately 34%-50% of patients with mild disease of less than one-year duration will have spontaneous resolution without the need for treatment, and approximately 14%-25% will have eventual total loss of hair on the scalp [2]. Almost a quarter of patients with AA have a family history of disease, suggesting a genetic predisposition [2]. Although AA is considered benign, psychological distress may result, disrupting patients’ self-esteem, and overall quality of life [3].

The exact pathologic process causing AA is unknown. It is hypothesized that there may be a loss of hair follicle (HF) immune privilege triggering the involvement of innate and adaptive components of the immune system. These interactions occur through oxidative stress ligands such as major histocompatibility complex (MHC) class I polypeptide-related sequence A (MICA), UL16-binding proteins (ULBP), and activated natural killer group 2D (NKG2D) receptors [4]. Patients will present clinically with ovoid patches of hair loss which may be present on the scalp, or elsewhere on the body. Patients who seek treatment will usually have scalp involvement. When the alopecia causes involvement of the entire scalp it is termed alopecia totalis, and when the entire body is involved, alopecia universalis. There may be nail changes such as pitting, ridging, and thickening [5]. About 70%-90% of hairs shift out of anagen phase, into catagen or telogen phases [6]. The terminal hairs are first to be involved, with the vellus hairs being involved as the disease progresses. Histopathologic changes are dependent on the duration of the disease and may be classified as early active (acute and subacute), or longstanding (chronic). During the early active stage, a peribulbar lymphocytic infiltrate will be visible, affecting the terminal HFs [5]. In longstanding or chronic stages, the lymphocytic infiltrate will involve the vellus hair as well, and most of the HFs will be in the catagen or telogen phase, with some being in the nanogen phase (miniaturized and rapidly cycling hairs with mixed features of the other three phases) [5-7].

A number of treatment options are available for hair regrowth in AA but none have been shown to be universally effective, or to completely rid the patient of disease. Few treatments have been verified by randomized controlled trials (RCTs), and the relatively high rate of spontaneous remission often clouds the interpretation of results. Topical steroids may be the first choice of treatment for mild disease limited to small areas. Results of RCTs vary, but some individuals appear to respond favorably [8-10]. Despite varying efficacy and the risk of developing folliculitis, topical steroids remain a first line choice due to their relative safety. Intralesional corticosteroid injections are effective, but are limited to use in small lesions or in cosmetically sensitive areas [11]. Skin atrophy is a common side effect in areas treated repeatedly, or with high doses [11]. Oral corticosteroids have also been used to treat AA and will produce regrowth in some patients [11]. However, prolonged treatment is needed to maintain the results, and does not justify the risk. Minoxidil, the treatment of choice for androgenic alopecia, has also been tested for the use in AA. One randomized controlled trial using topical minoxidil for the treatment of AA has demonstrated improved induction of hair growth over placebo in patients with mild disease [12], however further trials failed to show any improvement [13-15]. Nonetheless, this may be an option for patients due to the safety of the treatment. Perhaps the most underutilized option, contact immunotherapy, was first described in the 1970s and remains one of the most effective treatments for AA [16]. Typically, a contact allergen is applied weekly until hair regrowth is identified, at which point frequency may be reduced as needed. The process is intensive, requiring weekly visits for 6-32 months, and there is a risk for adverse effects including lymphadenopathy, severe dermatitis, and uncommonly severe urticaria and vitiligo [17-18]. There have also been concerns that several of the agents used are mutagenic [19-20]. Other treatment options including immunomodulators, low-level laser/light, photochemotherapy (psoralen plus ultraviolet A) [11], sulfasalazine, and others have been tested and showed mixed results [11]. Given the shortcomings of current treatments, new treatment modalities are currently being researched with a goal to improve results while minimizing adverse effects.

## Review

Methods

A literature search was performed in June of 2018 to identify publications involving fractional laser and microneedling used for the treatment of AA. PubMed was searched with the following terms: fractional laser OR microneedling OR microneedle OR micro needling AND alopecia. Abstracts of the 40 results, and pertinent references, were reviewed for inclusion. All trials, published in English, utilizing fractional lasers or microneedling for the treatment of AA were included.

Results

A total of six laser and two microneedling studies consisting of small prospective and retrospective studies, and case reports, were reviewed (Table 1). Fifty-seven patients, both males (n=29) and females (n=28), with AA were included.

**Table 1:** Summary of skin resurfacing studies for the treatment of alopecia areata *Nd:YAG: neodymium-doped yttrium aluminum garnet.

Author, year	Resurfacing technique	Additional treatment(s)	Study Design	Laser/needling settings	Number of sessions	Interval	Number of Subjects	Alopecia type	Adverse effects	results
Yalici-Armagan et al., 2016 [21]	CO_2_ laser (10600 nm) Nd:YAG* laser (1064 nm)	None	Prospective, split lesion study	10-45 mJ/cm^2^, 75-100 spots/cm^2^ 10 J/cm^2^, 30 millisecond pulse	3-6	2-4 weeks 2-8 weeks	32 (19 male, 13 female)	Alopecia areata	Pain	No improvement
Issa et al., 2016 [22]	CO_2_ laser (10600 nm)	Topical corticosteroid	Prospective study	60 mJ/spot, 100 spots/cm^2^, 2 passes	1-6	3 weeks	5 (1 male, 4 female)	Alopecia areata	Mild burning pain	Improvement in all patients
Eckert et al., 2016 [23]	Erbium glass laser (1550 nm)	None	Retrospective series	Variable	2-3	3-6 weeks	5 (2 male, 3 female)	Alopecia areata	Pain	Improvement in all treated lesions
Cho et al., 2013 [24]	CO_2_ laser (10600 nm) Erbium glass laser (1550 nm)	Variable	Retrospective series	30-50 mJ/spot, 150 spots/cm^2^ 6-8 mJ/spot, 300 spots/cm^2^	2-3	3-6 weeks	3 (1 male, 2 female)	Ophiasis	Pain, crusting, scaling, erythema, and edema	2 of 3 patients demonstrated improvement
Tsai, 2011 [25]	Erbium glass laser (1550 nm)	Intralesional corticosteroid injection	Split scalp case report	Not reported	12	1 week	1 male	Alopecia areata	None	Improvement vs steroid alone
Yoo et al., 2010 [26]	Erbium glass laser (1550 nm)	None	Case report	10-15 mJ/spot, 300 spots/cm^2^, 2 passes	24	1 week	1 male	Alopecia areata	None	Complete regrowth in treated lesions
Mysore et al., 2014 [27]	Microneedle dermaroller	Topical corticosteroids	Case series		3	3 weeks	2 males	Alopecia areata	None	Improvement
Yoo et al., 2010 [28]	1 mm microneedle roller	Topical methyl 5-aminolevulinate acid and photodynamic therapy with red light	Split lesion case series		3	4 weeks	8 (2 male, 6 female)	Alopecia areata	Mild pain, and erythema	No improvement

Fractional non-ablative Erbium glass [23-26], fractional ablative CO_2_ [21-22,24], and non-ablative neodymium-doped yttrium aluminum garnet (Nd:YAG) lasers [21] have been used for treatment of AA. Both the CO_2_ and Erbium glass lasers have a chromophore of water and effectively vaporize or coagulate tissue upon which they act [29]. The non-ablative Nd:YAG laser has a target chromophore of melanin and can penetrate effectively into deeper tissue [30].

A prospective split-lesion trial examining the effect of the CO_2_ laser and Nd:YAG laser, without topical drug application, on 19 men and 13 women with AA failed to show an increase in mean hair density [21]. The study subjects had three affected areas allocated to treatment with either Nd:YAG or CO_2_ laser, or no treatment. In a prospective series, investigators paired a single administration of topical corticosteroid with CO_2_ laser treatment [22]. All five patients exhibited clinical improvement, sustained at 12-month follow-up. One patient had additional control patches treated with laser alone which were noted to have no improvement. In a retrospective review of three patients with AA (ophiasis), treated with a combination of CO_2_ and Erbium glass lasers, two saw moderate to marked improvement, while one did not show any change [24]. Of the patients with improvement, one was treated with concurrent intrafollicular corticosteroid injections.

Two case reports and one additional case series using the Erbium glass laser have been identified and included for review in our study [23,25-26]. A case of complete regrowth of areas of alopecia areata occurred in a male patient after 24 treatment sessions, at weekly intervals [26]. No other treatments were given during this time and the patient maintained complete hair regrowth at six-month follow-up. Another case of a 28-year-old man with alopecia totalis treated with Erbium glass laser in conjunction with intralesional corticosteroid has been reported [25]. After 12 weekly treatment sessions, it was noted that the site treated with combination therapy was greatly improved over corticosteroid injection alone. In a case series of five patients with AA treated with Erbium glass laser, patients had full, or near full regrowth at three-months with no recurrence of any of the treated patches at two-to four-year follow-up [23].

Only two identified reports evaluated the efficacy of microneedling devices in the treatment of AA. In a series of eight patients, microneedling in conjunction with methyl 5-aminolevulinate and photodynamic therapy was ineffective for the treatment of AA [28]. Conversely, in a case series of two men with AA, a combination of microneedling and topical corticosteroids was found to be effective at restoring hair growth [27].

Discussion

In the reviewed studies, the treatments were tolerated by patients and the adverse effects were limited to pain, and transient erythema, edema, and pruritus. There is very little evidence to suggest that microneedling or treatment with fractional laser devices alone aids in the restoration of hair growth in patients with AA. The largest study to date examined the effects of fractional ablative CO_2_ laser and non-ablative Nd:YAG laser in a prospective split-lesion study of 32 patients [21]. There was no notable difference found between treatment and non-treatment sites. One report showed mixed results [24], while another showed positive effects [26]. These mixed results can be attributed to the natural remitting-relapsing course of the disease. However, one case series of five patients noted improvement with no recurrence of those lesions after two to four years [23]. These results would be difficult to explain solely by disease course. As the laser type and settings remain heterogeneous throughout these studies, it is possible that the laser settings used for these five patients can produce favorable results. Given the poor evidence for the use of these techniques alone, the utility of the fractional laser and microneedling devices for the treatment of AA is probably centered around the ability of these treatments to enhance drug delivery to the target tissue. All studies using topical corticosteroids in conjunction with laser or microneedling treatments showed improvement in hair growth. However, other than a single case report [27], there have been no comparative studies to suggest that combined therapy is more effective than topical steroids alone. There are significant limitations to this review. Many of the examined studies are case reports or small series. Given the heterogeneity of patients and treatment types, it is difficult to draw a substantial conclusion. Many treatment variables remain untested and unknown. Laser type, settings, treatment frequency, and topical therapeutic options require further examination.

One of the proposed mechanisms by which microneedling and fractional lasers may induce hair regrowth is by altering the microenvironment and causing changes to the local immune cells [31]. Through the release of various chemokines, there is potential to displace the perifollicular infiltrate to other areas of the dermis and epidermis. It has also been proposed that laser light can trigger apoptosis of lymphocytic cells, relieving the immune-mediated destruction of the follicles [24,26]. Alternatively, the paradoxical hair growth seen in cases of laser hair removal [32-33] may be a mechanism by which hair restoration could occur in patients with AA. It is hypothesized that the stimulation, and incomplete destruction of the HF can stimulate a regenerative response triggering the HF to enter anagen. Similarly to the proposed mechanism of minoxidil, enhanced blood flow to the HF caused by microtrauma could enhance hair growth. Additionally, these modalities have both been shown to enhance dermal drug delivery through the creation of columns of tissue injury, acting as a conduit for the passage of topically applied substances [34-35].

## Conclusions

Microneedling and fractional laser therapy have been shown to have minimal risk of severe or lasting adverse effects within a small group of patients with AA. Several mechanisms may be responsible for the induction of hair growth after skin resurfacing procedures, though the precise mechanisms responsible remain to be determined. The paucity of clinical data for these new treatment modalities does not allow one to make any definitive conclusions regarding their efficacy. There is potential for the therapies to be effective when combined with topical drug administration due to enhanced drug delivery. Further examination in the form of randomized or split-lesion studies is needed to determine whether this approach is clinically viable.
